# Experimental Researches in Cerebral Pathology

**Published:** 1874-01

**Authors:** 


### Experimental Researches in Cerebral Pathology.

The following is Professor Ferrier's summary of his very important "Experimental Researches in Cerebral Physiology and Pathology," which appeared originally in the *British Medical Journal* for April 26, 1873, and subsequently with a full account of the experiments in the West Riding Lunatic Asylum Medical Reports, vol. iii. There is no doubt that those experiments open up a most important field and mode of research. To be able to stimulate directly limited portions of the brain in a living animal is a great advance of anything as yet attempted in investigation of cerebral function. It is not only what Professor Ferrier's experiments prove, but what they suggest, and will undoubtedly lead to, that gives them their superlative interest to all students of brain function.

1. The anterior portions of the cerebral hemispheres are the chief centers of voluntary motion and the active outward manifestations of intelligence.

2. The individual convolutions are separate and distinct centers; and in certain definite groups of convolutions (to some extent indicated by the researches of Fritsch and Hitzig) and in corresponding regions of non convoluted brains, are localised the centers for the various movements of the eyelids, the face, the mouth (and tongue), the ear, the neck, the hand, foot and tail. Striking differences corresponding with the habits of the animals are found in the differentiation of the centers. Thus the centers for the tail in dogs, the paw in cats, and the mouth in rabbits, are highly differentiated and pronounced.

3. The action of the hemispheres is in general crossed; but certain movements of the mouth, tongue and neck are bilaterally co-ordinated from each cerebral hemisphere.

4. The proximate causes of the different epilepsies are, as Dr. Hughlings Jackson supposes, discharging lesions of the different centers in the cerebral hemispheres. The affection may be limited artificially to one muscle, or group of muscles, or may be made to involve all the muscles represented in the cerebral hemispheres, with foaming at the mouth, biting the tongue, and loss of consciousness. When induced artificially in animals, the affection as a rule first invades the muscles most in voluntary use, in striking harmony with the clinical observations of Dr. Hughlings Jackson.

5. Chorea is of the same nature as epilepsy, dependent on momentary (and successive) discharging lesions of the individual cerabral centers. In this respect Dr. Hughlings Jackson's views are again experimentally confirmed.

6. The corpora striata have crossed action and are centers for the muscles of the opposite side of the body. Powerful irritation of one causes rigid pleurosthotonos, the flexors predominating over the extensors.

7. The optic thalamus, fornix, hippocampus major, and convolutions grouped around it, have no motor signification (and are probably connected by sensation).

8. The optic lobes or corpora quadrigemina, besides being concerned with vision and the movements of the iris, are centers for the extensor muscles of the head, trunk and legs. Irritation of these centers causes rigid opisthotonos (and trismus).

9. The cerebellum is the co-ordinating center for the muscles of the eyeball. Each separate lobule (in rabbits) is a distinct center for special alterations of the optic axes.

10. On the integrity of these centers depends the maintenance of the equilibrium of the body.

11. Nystagmus, or oscillation of the eyelids, is an epileptiform affection of the cerebellar oculo-motorial centers.

12. These results explain many hitherto obscure symptoms of cerebral disease, and enable us to localise with greater certainty many form of cerebral lesion.—*The Clinic.*

